# Thymol Preserves Spermatogenesis and Androgen Production in Cisplatin-Induced Testicular Toxicity by Modulating Ferritinophagy, Oxidative Stress, and the Keap1/Nrf2/HO-1 Pathway

**DOI:** 10.3390/biom15091277

**Published:** 2025-09-03

**Authors:** Amira M. Badr, Sheka Aloyouni, Yasmin Mahran, Hanan Henidi, Elshaymaa I. Elmongy, Haya M. Alsharif, Aliyah Almomen, Sahar Soliman

**Affiliations:** 1 Department of Pharmacology and Toxicology, College of Pharmacy, King Saud University, P.O. Box 22452, Riyadh 11495, Saudi Arabia; amibadr@ksu.edu.sa; 2 Department of Pharmacology and Toxicology, College of Pharmacy, Ain Shams University, Cairo 11566, Egypt; 3 Genetics Section, Research Department, Natural and Health Sciences Research Center, Princess Nourah bint Abdulrahman University, P.O. Box 84428, Riyadh 11671, Saudi Arabia; syaloyouni@pnu.edu.sa; 4 Research Department, Natural and Health Sciences Research Center, Princess Nourah bint Abdulrahman University, P.O. Box 84428, Riyadh 11671, Saudi Arabia; hahenidi@pnu.edu.sa; 5 Department of Pharmaceutical Chemistry, Faculty of Pharmacy, Helwan University, Cairo 11795, Egypt; shaymaa.taha@pharm.helwan.edu.eg; 6 College of Pharmacy, King Saud University, P.O. Box 22452, Riyadh 11495, Saudi Arabia; hayaaalsharif0@gmail.com; 7 Department of Pharmaceutical Chemistry, College of Pharmacy, King Saud University, Riyadh 11451, Saudi Arabia; alalmomen@ksu.edu.sa; 8 Department of Physiology and Pharmacology, College of Osteopathic Medicine, Sam Houston State University, Conroe, TX 77304, USA

**Keywords:** cisplatin, thymol, ferritinophagy, testicular toxicity, steroidogenesis, Nrf2

## Abstract

Cisplatin (CDDP) is a widely used chemotherapeutic agent, but its off-target toxicity, including testicular damage, limits clinical use. Bioactive compounds may help mitigate chemotherapy-induced reproductive toxicity. This study investigates thymol’s role in modulating ferritinophagy to preserve reproductive function and steroidogenesis. Male Wistar rats were randomized to control, CDDP, thymol, or CDDP + thymol groups. Thymol (60 mg/kg) was given orally for 14 days, and CDDP (8 mg/kg) was administered intraperitoneally on day 7. Testicular function was assessed through hormonal analysis, sperm evaluation, and histopathology. Ferritinophagy, oxidative stress, and inflammatory markers were assessed to elucidate thymol’s chemoprotective mechanisms. Thymol co-administration preserved steroidogenesis, restored sperm quality, and maintained testicular architecture in CDDP-treated rats. Thymol suppressed ferritinophagy, reducing iron overload and mitigating reactive oxygen species (ROS)-induced cellular damage. Additionally, thymol activated the Keap1/Nrf2/HO-1 pathway, enhancing antioxidant defenses while downregulating inflammatory mediators (TNF-α, IL-6). Additionally, thymol enhanced CDDP’s selectivity toward cancer cells while reducing its toxicity to normal cells. This study provides evidence that thymol modulates ferritinophagy to attenuate CDDP-induced testicular toxicity, helping preserve reproductive function via regulation of iron homeostasis. These findings highlight thymol’s potential as an adjunct therapy to mitigate chemotherapy-associated reproductive damage while maintaining CDDP’s anticancer efficacy.

## 1. Introduction

CDDP, despite being a cornerstone therapy for various malignancies, has its therapeutic utility often limited by significant toxicities [1,2,3,4,5,6,7]. Testicular toxicity is of particular concern due to its implications for fertility, hormonal regulation, and long-term reproductive health [8,9,10]. The underlying mechanisms of CDDP-induced toxicity are under investigation to enhance the drug’s safety and therapeutic utility.

Spermatogenesis is an intricate and highly regulated process involving germ cell proliferation, differentiation, and maturation, relying on a fine balance of hormonal signaling and cellular integrity within the seminiferous tubules [11]. CDDP-induced gonadotoxicity leads to azoospermia, testicular damage, and long-term failure of spermatogenesis [12,13], driven by oxidative stress, inflammation, and apoptosis. Similar mechanisms drive a spectrum of toxicities, including neurotoxicity and nephrotoxicity [5,14,15], compromising multiple organ systems.

The interplay between iron dysregulation and oxidative stress is a key driver of CDDP-induced cellular injury. Very recently, studies have attempted to understand the role of ferritinophagy-mediated iron release in CDDP-induced oxidative damage [16,17,18]. In this process, NCOA4 (nuclear receptor coactivator 4) selectively binds to ferritin, directing it to lysosomal degradation and liberating stored iron into the cytosol. This influx of free iron expands the labile iron pool, fueling reactive oxygen species (ROS) generation via Fenton chemistry [19,20]. In the Fenton reaction, ferrous iron (Fe^2+^) reacts with hydrogen peroxide (H_2_O_2_) to produce hydroxyl radicals, highly reactive species that initiate lipid peroxidation and damage cellular macromolecules [21]. The accumulation of ROS drives lipid peroxidation, leading to membrane instability and ferroptosis, an iron-dependent form of regulated cell death [22]. Unlike apoptosis, which is caspase-mediated, ferroptosis is characterized by oxidative injury, iron overload, and lipid peroxidation-driven cytotoxicity [23].

To counteract oxidative stress, cells activate the Nrf2/Keap1 pathway, a key regulator of antioxidant defense. Under basal conditions, Nrf2 (nuclear factor erythroid 2–related factor 2) is sequestered by Keap1 (Kelch-like ECH-associated protein 1), targeting it for ubiquitination and proteasomal degradation [24,25]. However, oxidative stress disrupts this interaction, allowing Nrf2 to escape Keap1-mediated degradation, translocate to the nucleus, and drive the expression of cytoprotective genes, including heme oxygenase-1 (HO-1) [26], which degrades heme into biliverdin, carbon monoxide, and free iron, thus contributing to antioxidant defense [27].

Thymol, a monoterpene phenol derived from the essential oils of aromatic plants such as *Thymus vulgaris*, has demonstrated a wide spectrum of biological activities, including antioxidant, anti-inflammatory, and antimicrobial effects, suggesting its relevance in protecting against chemotherapy-induced organ toxicities [28,29]. In experimental models, thymol has shown protective effects against hepatotoxicity and nephrotoxicity through mechanisms such as reducing ROS production, preserving cellular architecture, and modulating inflammatory markers [29,30,31]. However, its role in combating CDDP-induced testicular toxicity remains to be identified.

By reducing ROS production and modulating inflammatory pathways, thymol may preserve the cellular environment necessary for spermatogenesis. Additionally, its ability to influence steroidogenesis and maintain hormonal balance could further support germ cell maturation and function [32]. Investigating thymol’s potential to counteract CDDP-induced disruptions in spermatogenesis offers a promising avenue for improving reproductive outcomes in cancer patients receiving CDDP as part of their chemotherapeutic regimen.

The present study investigates whether thymol mitigates CDDP-induced testicular damage by modulating oxidative stress, inflammatory responses, and ferritinophagy. We hypothesize that thymol exerts chemoprotective effects through activation of the Keap1/Nrf2/HO-1 pathway and suppression of NCOA4-mediated ferritinophagy, thereby preserving spermatogenesis and steroidogenesis. The objectives are to evaluate the structural and functional impact of thymol on testicular tissue, characterize its molecular mechanisms of action, and determine whether thymol interferes with or enhances the anticancer efficacy and selectivity of CDDP. This work provides preclinical evidence supporting thymol’s potential as a fertility-preserving adjunct during chemotherapy.

## 2. Materials and Methods

### 2.1. Molecular Modeling Study

The crystallized protein structure of HO-1 was retrieved from the Protein Data Bank (PDB ID: 3HOK), along with its co-crystallized ligand “2-({[(2R,4S)-2-[2-(4-chlorophenyl)ethyl]-2-(1H-imidazol-1-ylmethyl)-1,3-dioxolan-4-yl]methyl}sulfanyl)-5-(trifluoromethyl)pyridine” assigned a ligand ID of Q80. The ligand was used to define the docking binding site. This structure was selected based on its high resolution, the presence of a co-crystallized ligand suitable for defining the active site (Supplementary Figures S1 and S2), and its established use in the literature [33,34].

The structure of acyl-CoA synthetase long-chain family member 4 (ACSL4) was obtained from Uniport (entry O60488) and downloaded as a 3D structure in PDB format from AlphaFold Database. A Ramachandran plot was generated for ACSL4 using Molprobity (Duke University, Durham, NC, USA), including six cases: one general and five specific cases involving glycine, preproline and proline (not shown). Docking interactions and binding pocket visualization for ACSL4 are provided in Supplementary Figures S3 and S4.

Thymol structure was sketched as a 2D structure in ChemSketch 2020.2 (ACD/Labs, Toronto, ON, Canada), converted to 3D using Open Babel 3.1.1 and saved in SDF format for use in UCSF Chimera 1.17.3 (University of California, San Francisco, CA, USA) and Biovia Discovery Studio 21.1.0.20298 (Dassault Systèmes, Vélizy-Villacoublay, France).

Ligands and protein targets were prepared using UCSF Chimera by adding hydrogen atoms and assigning Gasteiger charges, followed by energy minimization. Gridbox was generated using AutoGrid 4.2.6 (The Scripps Research Institute, La Jolla, CA, USA) allocated at the macromolecule center. For HO-1 the grid box was centered on the co-crystallized ligand with site sphere coordinates X = 14.17, Y = −1.89, Z = 34.12. For ACSL4, the grid encompassed the full protein with coordinates X = 2.37, y = −1.266, Z = −8.08 with a threshold of 2.5, grid spacing of 0.5, and grid angle of 90°.

Docking was performed using AutoDock Vina 1.2.5 (The Scripps Research Institute, La Jolla, CA, USA). The results were saved in PDBQT format and visualized using Biovia Discovery Studio.

### 2.2. In Vivo Study

#### 2.2.1. Animal Studies

Animal studies were performed in accordance with procedures approved by the Research Ethics Committee at Institutional Review Board, King Abdullah Bin Abdulaziz University Hospital, Riyadh, Saudi Arabia (IRB number: 24-0146). Adult (7–8 week old) male Wistar rats, weighing between 150–200 g, were obtained from the Experimental Animal House, Faculty of Pharmacy, King Saud University. Rats were singly housed under standard conditions (25 °C, 60% humidity, and a 12-h light-dark cycle) with unrestricted access to water and a standard diet containing no less than 20% protein, 5% fiber, 3.5% fat, 6.5% ash, and a vitamin mixture. A one-week acclimatization period was provided prior to experimental procedures.

#### 2.2.2. Experimental Design

Animals were randomly assigned to one of four treatment groups (8 animals per group) by a blinded researcher: Control (C), CDDP, Thymol, or a combination of CDDP and thymol (CDDP + Thymol). The Control group received the vehicle solution (1% tween in normal saline) once daily via oral gavage for 14 days and intraperitoneal injection of saline on day 7. The CDDP group received a single intraperitoneal injection of CDDP (8 mg/kg) on day 7 [35]. The Thymol group received thymol (60 mg/kg) in the vehicle once daily via oral gavage for 14 days as previously described [29,31]. The Combination group received thymol (60 mg/kg) in the vehicle once daily via oral gavage for 14 days and a single intraperitoneal injection of CDDP (8 mg/kg) on day 7. The 60 mg/kg thymol dose was selected based on previous studies demonstrating its efficacy and safety in rodent models without inducing toxicity [36,37,38]. This dose has been widely used in similar contexts involving inflammation and oxidative stress. The CDDP dose (8 mg/kg) was selected based on well-established preclinical models, where 7–8 mg/kg of CDDP is commonly used to induce testicular toxicity [39,40,41,42].

Animals were monitored daily for weight changes and general health. On day 15, all animals were euthanized using carbon dioxide (CO_2_), sacrificed, and the blood was immediately collected via cardiac puncture using a sterile syringe. Blood samples were transferred into tubes, allowed to clot, and centrifuged at 3000 rpm for 30 min at 4 °C to separate the serum, which was stored at −80 °C for subsequent analysis.

#### 2.2.3. Testicular Tissue Collection, Preparation and Sperm Analysis

Following decapitation, testes were immediately excised, weighed, and washed with phosphate-buffered saline (PBS) to remove blood and debris. For sperm analysis, the cauda epididymis was dissected, sliced, and immersed in 1 mL of PBS (pH 7.4) maintained at 37 °C. The sperm suspension was diluted at a 1:20 ratio in PBS (pH 7.2), and total sperm count was determined using a hemocytometer. Sperm morphology was assessed on hematoxylin and eosin-stained slides under 1000× magnification.

Testicular tissues were divided into two parts: the first part was fixed in 10% neutral buffered formalin for 24 h for histological and immunohistochemical analyses. The second portion was snap-frozen in liquid nitrogen and stored at −80 °C for molecular and biochemical assays.

#### 2.2.4. Histological and Immunohistochemical Analysis

Fixed testicular tissues were processed into paraffin blocks and sectioned at 5 µM thickness. Hematoxylin and eosin (H&E) staining was used for histological assessment, Spermatogenesis was assessed using Johnsen’s scoring system, which rates germ cell development on a scale from 1 to 10, where 10 indicates full spermatogenesis and 1 reflects complete absence of seminiferous epithelium. For each animal, at least 50 randomly selected tubules were examined under light microscopy, and a mean Johnsen score was calculated. This scoring system is widely used to assess testicular damage and has been applied in previous studies [43]. Additionally, seminiferous tubule dimensions, including area and volume, were quantitatively analyzed as described previously [44].

Keap 1, Nrf2 and HO-1 expressions were assessed using immunohistochemical analysis. Antigen retrieval on deparaffinized sections was performed by heating in 0.05 M citrate buffer (pH 6.8). Endogenous peroxidase activity was quenched with 0.3% hydrogen peroxide, followed by blocking with a protein-blocking solution to reduce non-specific binding. Sections were incubated overnight at 4 °C with a primary anti-keap1 (Abcam (Cambridge, UK), 1:200 dilution), anti-Nrf2 (Proteintech, Munich, Germany, 1:300 dilution) and anti-HO-1 antibody (Abcam, ab13248; 1:200 dilution). After PBS washes, a secondary antibody HRP Envision kit (DAKO) was applied 20 min; washing by PBS and incubated with diaminobenzidine (DAB) for 10 min. Washing by PBS then counter staining with hematoxylin, dehydrated and clearing in xylene then cover slipped for microscopic analysis. The slides were subsequently examined under a light microscope, and representative images were captured for analysis Using Full HD microscopic camera operated by Leica application module for tissue sections analysis operated by examiner histologist (Leica Microsystems GmbH, Wetzlar, Germany). At least 6 random non overlapping fields from each sample were scanned and analyzed for calculation of mean immunohistochemical expression levels of Keap1, Nrf2 and HO-1 in germinal epithelium of immunostained tissue sections according to the method adapted from Elsayed et al., 2022 [45].

#### 2.2.5. Quantification of Serum Testosterone and Luteinizing Hormone (LH)

Serum concentrations of testosterone and LH were quantified using highly sensitive enzyme-linked immunosorbent assay (ELISA) kits. The testosterone ELISA kit (Cat. #K7418-100) was purchased from BioVision (Milpitas, CA, USA), and the LH ELISA kit (Cat. #RAT11459) was obtained from AFG Scientific (Northbrook, IL, USA). Assays were performed according to the manufacturers’ protocols, and absorbance was measured at 450 nm using a BioTek ELx808LBS microplate reader (BioTek Instruments, Winooski, VT, USA).

#### 2.2.6. Assessment of Oxidative Stress Biomarkers

Oxidative stress in testicular tissues was evaluated by measuring malondialdehyde (MDA) levels, a marker of lipid peroxidation. Homogenized testicular tissues were prepared and analyzed using the MDA Colorimetric Assay Kit (Invitrogen, Carlsbad, CA, USA), per the manufacturer’s instructions. The absorbance of the resulting adduct was measured at 532 nm using a BioTek ELx808LBS microplate reader.

Superoxide dismutase (SOD) activity was measured in testicular homogenates using the pyrogallol autoxidation method described by Gao et al. [46]. The assay was performed as per the specified protocol, and the activity was quantified spectrophotometrically.

Glutathione peroxidase (GPx) activity was assessed in homogenized testicular tissues using a GPx ELISA kit (Cat. No. MBS1600242, MyBioSource, San Diego, CA, USA) according to the manufacturer’s instructions.

#### 2.2.7. Assessment of Inflammatory Markers

The expression levels of tumor necrosis factor-alpha (TNF-α) and interleukin-6 (IL-6) were measured in homogenized testicular tissues using commercially available ELISA kits (TNF-α: Cat. No. RAB0480 Sigma-Aldrich, St. Louis, MI, USA; IL-6: Cat. No. ab234570, Abcam, Waltham, MA, USA). The assays were performed according to the manufacturer’s instructions, and the concentrations of the inflammatory markers were quantified spectrophotometrically.

#### 2.2.8. Assessment of Ferrous Level in Testis

The testicular ferrous level was quantified using a colorimetric assay kit (BioDiagnostic, Giza, Egypt), following the manufacturer’s protocol. Results were expressed as μg iron per gram of wet tissue.

#### 2.2.9. Quantification of mRNA Expression by Real-Time Polymerase Chain Reaction (RT-PCR) for Steroidogenesis and Ferritinophagy- Related Genes

The genes analyzed by RT-PCR were selected based on their involvement in key biological pathways disrupted during cisplatin-induced testicular toxicity. These included markers of steroidogenesis—StAR (steroidogenic acute regulatory protein), 3β-HSD (3β-hydroxysteroid dehydrogenase), and 17β-HSD (17β-hydroxysteroid dehydrogenase)—which are essential enzymes regulating testosterone biosynthesis in Leydig cells. To investigate the role of ferroptosis and iron metabolism, we also assessed TFRC (transferrin receptor 1), SLC7A11 (cystine/glutamate antiporter), ACSL4 (acyl-CoA synthetase long-chain family 4), and NCOA4 (nuclear receptor coactivator 4). These genes were selected due to their established roles in regulating iron uptake, lipid peroxidation, glutathione metabolism, and ferritinophagy, all of which are central to ferroptotic cell death. Together, these targets provided a comprehensive molecular profile of steroidogenic impairment and ferroptosis activation in response to CDDP and thymol treatment.

RNA extraction from tissue samples was performed using QIAamp RNeasy Mini kit (Cat. No. 74104; Qiagen, Germany). A volume of 200 µL of each sample was mixed with 600 µL RLT buffer containing 10 μL β-mercaptoethanol per 1 millileter and incubated at room temperature for 10 min. Equal volume of 70% ethanol was added to the cleared lysate, and RNA purification was completed according to the manufacturer’s protocol. Primers (Table 1) and the reference gene Glyceraldehyde 3-phosphate dehydrogenase (GAPDH) were supplied by HavenSci (Innovation Center, KAUST, Thuwal 23955, Saudi Arabia). Primers were used in a 20-µL reaction mixture containing 10 µL of 2× HERA SYBR^®^ Green RT-qPCR Master Mix (Cat. No. WF10304001; Willowfort, Nottingham, UK), 1 µL of RT Enzyme Mix (20×), 1 µL each of forward and reverse primers (20 pmol), 3 µL of nuclease-free water, and 5 µL of RNA template.

Quantitative RT-PCR was performed using a QuantStudio^®^ RT-PCR Instrument (96-well, 0.2 mL block; Applied Biosystems, Thermo Fisher Scientific Inc., Waltham, MA, USA). Gene expression levels were analyzed using the ΔΔCt method, with Ct values normalized to the reference gene and compared to the positive control group, as described by Yuan et al. (2006). Relative expression levels were calculated using the 2^−ΔΔCt^ formula [47].

#### 2.2.10. Western Blot Analysis

The expression of SOD2, CD71 (transferrin Receptor 1 (TfR1), and GPX4 protein expression in testicular tissues was carried out using western blot analysis as previously described [15]. Band densities were quantified using ImageJ 1.53 software (National Institutes of Health, Bethesda, MD, USA). Data were expressed as fold change relative to the control group.

### 2.3. In Vitro Studies

The non-tumorigenic cell line (MCF-10A) and the breast cancer cell line (MCF-7) were obtained from the Natural and Health Sciences Research Centre in Riyadh, Saudi Arabia. Both cell lines were maintained in a humidified incubator at 37 °C with 5% CO_2_ in DMEM medium supplemented with 10% fetal bovine serum (FBS), 100 U/mL penicillin, and 100 μg/mL streptomycin. Cells were routinely trypsinized and passaged when they reached 80–90% confluence, with approximately 100,000–150,000 cells seeded per passage, following standard protocols.

#### 2.3.1. Cell Viability Assay

The anticancer effect of thymol, CDDP, and their combination was assessed using the MTT cell viability assay. MCF-7 and MCF-10A cells were seeded at a density of 5000 cells per well in 96-well plates and allowed to adhere for 24 h. Cells were then treated for 72 h with serial dilutions of CDDP, thymol, or their combination at concentrations ranging from 100 µM to 0.01 µM. After treatment, the medium was replaced with 20 μL of 20 mM MTT solution dissolved in PBS, and the cells were incubated at 37 °C with 5% CO_2_ for 3 h. Following incubation, the MTT solution was carefully removed, and 100 μL of DMSO was added to solubilize the violet formazan crystals. Cell viability was expressed relative to untreated control cells. Data were plotted using GraphPad Prism software, and IC_50_ values were calculated using a nonlinear regression (curve fit) model.

#### 2.3.2. Selectivity Index (SI)

The cytotoxic effect on MCF-10A cells was assessed to determine whether the combination improves CDDP’s selectivity for cancer cells. The selectivity ratio was determined using non-malignant MCF-10A cells and cancerous MCF-7 cells, according to the formula: SI = IC_50_ of normal cells/IC_50_ of cancer cells.

### 2.4. Statistical Analysis

All data were analyzed using GraphPad Prism version 10.2.2 (San Diego, CA, USA). Results are presented as mean ± standard deviation (SD). Comparisons between groups were performed using one-way analysis of variance (ANOVA) followed by Tukey-Kramer post hoc multiple comparison test. A *p*-value of <0.05 was considered statistically significant.

## 3. Results

### 3.1. Molecular Docking Analysis Reveals Thymol’s Interaction with Key Protective Pathways

To elucidate the mechanisms underlying thymol’s gonadoprotective effects, molecular docking simulations were performed. We sought to explore its interactions with proteins implicated in oxidative stress, inflammation, and ferritinophagy, key pathways involved in cisplatin-induced injury [9,12,14,16,42,48,49,50,51,52]. This approach was guided by our hypothesis and relevant literature, and was intended to help narrow the scope of subsequent mechanistic investigations.

**Figure 1:**
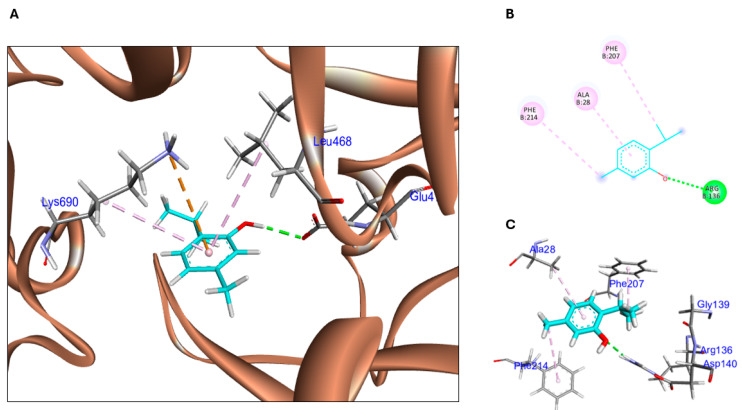

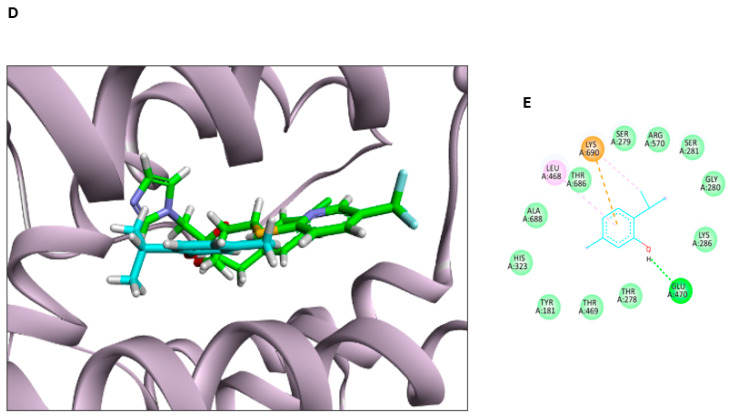
Molecular docking of thymol at the binding sites of HO-1 and ACSL4. (**A**) Thymol (blue sticks) superimposed on the co-crystallized ligand QC-80 (green sticks) within the HO-1 binding pocket. (**B**) Two-dimensional interaction diagram of thymol within HO-1, showing hydrogen bonds (green dashed lines) and hydrophobic interactions (pink dashed lines). (**C**) Three-dimensional interaction diagram of thymol with key residues in the HO-1 binding pocket. Hydrogen bonds are shown as green dashed lines, and hydrophobic interactions are shown as pink dashed lines. (**D**) Three-dimensional docking pose of thymol (blue sticks) within the ACSL4 binding site. (**E**) Two-dimensional interaction diagram of thymol and ACSL4, highlighting hydrogen bonds (green dashed lines), hydrophobic interactions (pink dashed lines), and electrostatic interactions (orange dashed lines). HO-1: heme oxygenase-1; ACSL4: acyl-CoA synthetase long-chain family 4.

The docked conformation of thymol within HO-1 is shown in Figure 1A–C, where thymol is superimposed on the co-crystallized ligand (QC-80), which was used to define the binding site near the secondary western pocket. The enzyme features two hydrophobic pockets: one located near the heme-binding pocket (northeastern) and the other in the distal region (western) [33]. The latter comprises a large primary cavity alongside a smaller secondary cavity. These pockets play a crucial role in influencing the potency of inhibitors by enhancing ligand-enzyme binding stability. Due to their flexibility, hydrophobic groups of varying sizes, such as aryl, biphenyl, or adamantyl moieties, can fit into the western and/or northeastern regions of the ligand. Additionally, modifications in the northeastern area have been shown to moderately increase HO-2 inhibition, whereas ligand occupation of the western pocket enhances HO-1 [53,54]. As illustrated in Figure 1B,C, thymol forms a conventional hydrogen bonding with Arg 136, in addition to three hydrophobic interactions: one between aromatic ring of thymol and Ala 28, and two between thymol alkyl side chain and aromatic rings of both Phe 207 and Phe 214.

Thymol was docked at ACSL4 (Figure 1D,E), where 97.7% (693/709) of all residues were in favored (98%) regions and 99.9% (708/709) of all residues were in allowed (>99.8%) regions. There was one outlier (phi, psi): 4 Lys (−58.7, 97.8) (Supplementary Figures S1 and S2). The 3D binding pose of thymol in ACSL4 is shown in Figure 1D, highlighting its fit within the active site pocket. The 2D interaction diagram is shown in Figure 1E, where thymol hydroxyl group interacts by a hydrogen bond with Glu 470 at a distance of 2.52Å, in addition to hydrophobic interactions with Leu 468 and Lys 690 at ~5 Å, and an electrostatic interaction between the aromatic ring of thymol and Leu 690 residue at ~4 Å (Supplementary Figures S3 and S4). Table 2 summarizes the binding energies and molecular interactions of thymol with HO-1 and ACSL4.

### 3.2. Thymol Attenuates CDDP-Induced Reductions in Body Weight Without Affecting Testis Weight

CDDP treatment is commonly associated with reductions in body weight, a hallmark of its systemic toxicity [42,55,56]. To assess the protective potential of thymol, we investigated whether its co-administration could mitigate these weight reductions in CDDP-treated animals. CDDP administration significantly reduced body weight compared to control animals. Thymol co-administration significantly countered this effect (Table 3). However, CDDP-induced reproductive toxicity was not associated with a reduction in testes weight, as shown in Table 3.

### 3.3. Thymol Restores Steroidogenic Gene Expression

**Figure 2:**
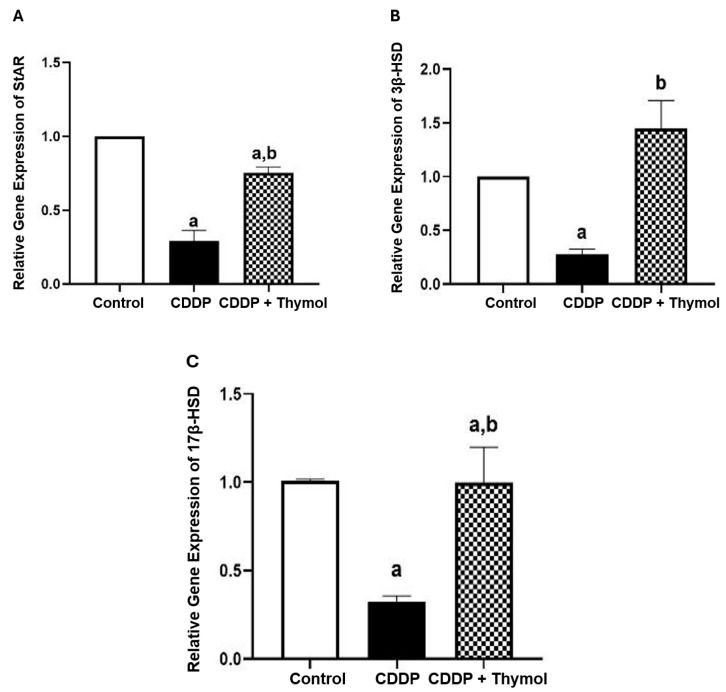
Thymol upregulated the mRNA expression of (**A**) StAR, (**B**) 3β-HSD, and (**C**) 17β-HSD in rats with CDDP-induced testicular damage. Data are expressed as mean ± SD (n = 3). a and b indicate significant differences compared to the control and CDDP groups, respectively (*p* < 0.05; ANOVA followed by Tukey-Kramer post hoc test). CDDP: cisplatin; StAR: steroidogenic acute regulatory protein; 3β-HSD: 3β-hydroxysteroid dehydrogenase; 17β-HSD: 17β-hydroxysteroid dehydrogenase.

To further elucidate thymol’s protective role in preserving hormonal function, we analyzed the mRNA expression of key steroidogenic genes using RT-PCR. CDDP treatment significantly downregulated the expression of critical genes involved in steroidogenesis, including StAR, 3β-HSD and 17β-HSD, which indicates impaired testosterone biosynthesis and disrupted Leydig cell function (Figure 2A–C).

Thymol co-treatment significantly upregulated the expression of these genes compared to the CDDP group. StAR relative expression showed a 2.6-fold increase (Figure 2A). The expression of 3β-HSD and 17β-HSD increased 5.2-fold, and 3.1-fold, respectively (Figure 2B,C).

This transcriptional upregulation was accompanied by partial recovery of serum testosterone and LH levels. Our results show that CDDP treatment significantly reduced serum testosterone levels by approximately 52% and LH levels by nearly 71% compared to the control group, indicating profound disruption in steroidogenesis and Leydig cell function (Table 3). Thymol co-treatment partially reversed these hormonal disturbances, increasing testosterone levels by about 59% and LH levels by nearly 174% relative to the CDDP-only group (Table 3). These results highlight thymol’s strong potential to mitigate CDDP-induced hormonal suppression and partially restore endocrine function toward normal levels.

### 3.4. Thymol Enhances Spermatogenic Recovery Despite Limited Structural Restoration in CDDP-Treated Animals

**Figure 3:**
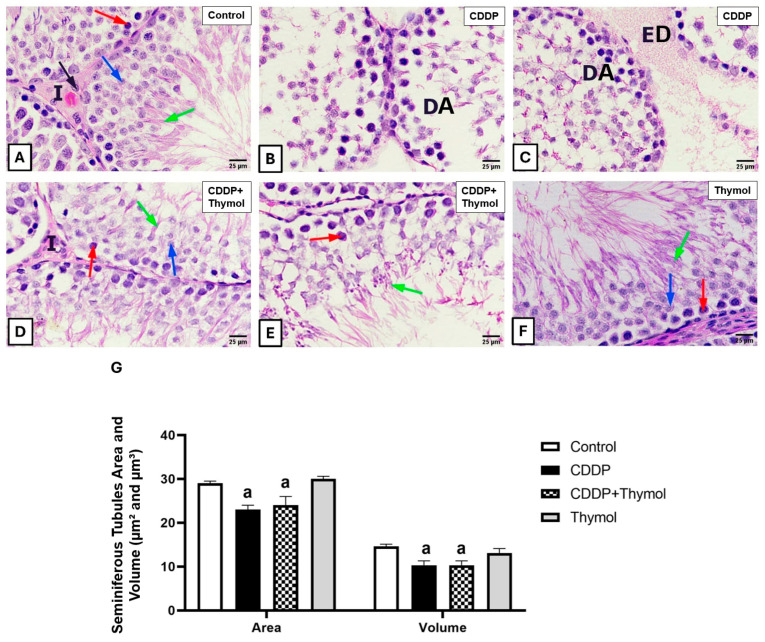
Thymol attenuates cisplatin-induced histological alterations in rat testes (H&E staining). (**A**) Photomicrograph of control testes showing normal seminiferous tubules filled with different stages of germ cells: primary spermatocyte (red arrow), secondary spermatocytes (blue arrows), spermatozoa (green arrow), Leydig cells (black arrow), and interstitial tissue (I). (**B**,**C**) Photomicrographs of testes tissues from rats in the CDDP group, showing wide degenerative area (DA), edema (ED), and sparse spermatocytes. (**D**,**E**) Photomicrographs of testes from the CDDP + thymol-treated group, showing preserved seminiferous tubule structure, interstitial tissue (I), primary spermatocyte (red arrow), secondary spermatocytes (blue arrows), and spermatozoa (green arrow). (**F**) Photomicrograph of testes from the thymol-only group, showing seminiferous tubules filled with various germ cell stages: primary spermatocyte (red arrow), secondary spermatocytes (blue arrows), and spermatozoa (green arrow). (**G**) Quantitative analysis of seminiferous tubule area and volume across different groups. Data are expressed as mean ± SD (n = 5). a indicates statistical significance compared to the control group (*p* < 0.05) using ANOVA followed by Tukey-Kramer as a post hoc test. CDDP: cisplatin; ED: edema; DA: degenerative area; I: interstitial tissue.

Histological examination of control testes revealed well-organized seminiferous tubules with intact epithelial structure and abundant interstitial Leydig cells (Figure 3A). All stages of spermatogenesis were present, yielding a Johnsen’s score of 10. Similarly, thymol-only treated animals maintained normal testicular histoarchitecture, with preserved spermatogenic layers and healthy interstitial tissue (Figure 3F).

In contrast, CDDP-treated testes exhibited marked histopathological alterations, including seminiferous epithelial disorganization, necrotic foci, and absence of late-stage germ cells (Figure 3B,C). These structural changes coincided with a significant 21% reduction in tubule area and a 30% decrease in volume relative to control, alongside a Johnsen’s score of 5.

Thymol co-administration partially reversed these CDDP-induced effects. Notable restoration of spermatogenic layers was observed (Figure 3D,E). The presence of primary and secondary spermatocytes, as well as mature spermatozoa, was evident, resulting in a Johnsen’s score of 9. However, this functional improvement was not accompanied by a comparable structural improvement. Tubule area and volume increased only marginally by 4% and 6%, respectively, compared to the CDDP group (Figure 3G).

### 3.5. Thymol Protects Against CDDP-Induced Azoospermia and Morphological Defects

**Figure 4:**
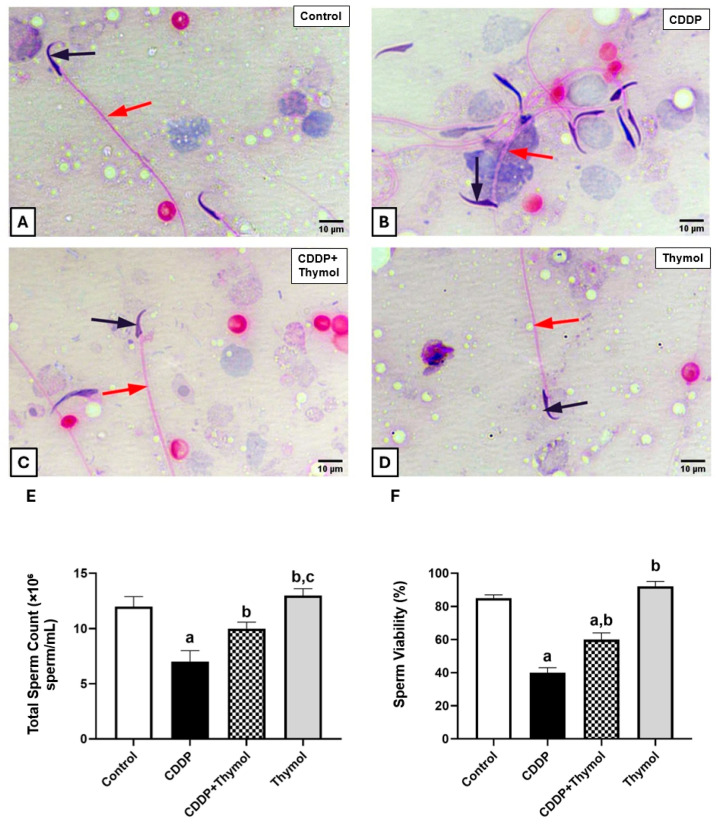
Effects of thymol and CDDP on sperm morphology, count, and viability in rats. (**A**) Control epididymal sperm showing normal rat sperm with a distinct head (black arrow) and straight tail (red arrow). (**B**) Epididymal sperm from the CDDP group displaying deformed sperm with distorted heads (black arrow) and bent tails (red arrow). (**C**) Sperm from the CDDP + thymol group showing partially deformed sperm with bent tails (red arrow) and slightly distorted head (black arrow). (**D**) Sperm from the thymol-only group displaying healthy morphology with a distinct clear head (black arrow), and straight tail (red arrow). (**E**) Total sperm count. (**F**) Percentage of sperm viability. Data are expressed as mean ± SD (n = 5). a, b, and c indicate statistical differences from the control, CDDP, and CDDP + thymol groups, respectively (*p* < 0.05) using ANOVA followed by Tukey–Kramer post hoc test. CDDP: cisplatin.

Morphological analysis of hematoxylin and eosin-stained epididymal sections revealed that sperm from the control and thymol-only groups exhibited typical healthy morphology, characterized by distinct hooked heads, straight tails, and intact cellular structures (Figure 4A,D). In contrast, the CDDP-treated group showed pronounced abnormalities, including deformed heads with flattened hooks, bent tails, and signs of degeneration, indicating substantial morphological damage (Figure 4B). Co-treatment with thymol preserved normal structural features, with intact heads and minimal abnormalities (Figure 4C).

In line with these morphological findings, CDDP administration significantly reduced total sperm count by approximately 42% compared to the control. Thymol co-administration partially restored sperm count to around 83% of control levels, while thymol-only treatment increased the count slightly above control values (Figure 4E).

Sperm viability followed a similar pattern. CDDP significantly reduced viability by 53%, from 85% in the control group to 40%. Thymol co-treatment improved viability to 60%, achieving a 71% recovery relative to control, whereas thymol alone elevated viability to 92% (Figure 4F).

### 3.6. Thymol Modulates Oxidative Stress and Inflammatory Markers Expression Altered by CDDP

To assess thymol’s potential antioxidant and anti-inflammatory effects in CDDP-induced testicular toxicity, we measured the levels of oxidative stress markers (MDA, SOD, GPX) and pro-inflammatory cytokines (TNF-α and IL-6). CDDP treatment resulted in a significant increase in MDA levels by approximately 89% compared to the control group, indicating increased lipid peroxidation. In parallel, SOD and GPX activities were significantly reduced by 45% and 39%, respectively, confirming a marked impairment of the antioxidant defense system.

Co-administration of thymol with CDDP significantly decreased MDA levels by 11% relative to the CDDP group, while SOD and GPX activities were significantly elevated by 35% and 55%, respectively.

Additionally, CDDP significantly increased TNF-α expression by 88% and reduced IL-6 by 42% relative to the control. Thymol co-treatment significantly reduced TNF-α by 33% and elevated IL-6 by 28% when compared to CDDP alone (Table 4). Collectively, these findings demonstrate that thymol mitigates CDDP-induced oxidative stress and inflammatory disturbances, likely contributing to its overall gonadoprotective effect.

### 3.7. Thymol Ameliorates CDDP-Mediated Suppression of the Keap1/Nrf2/HO-1 Pathway

**Figure 5:**
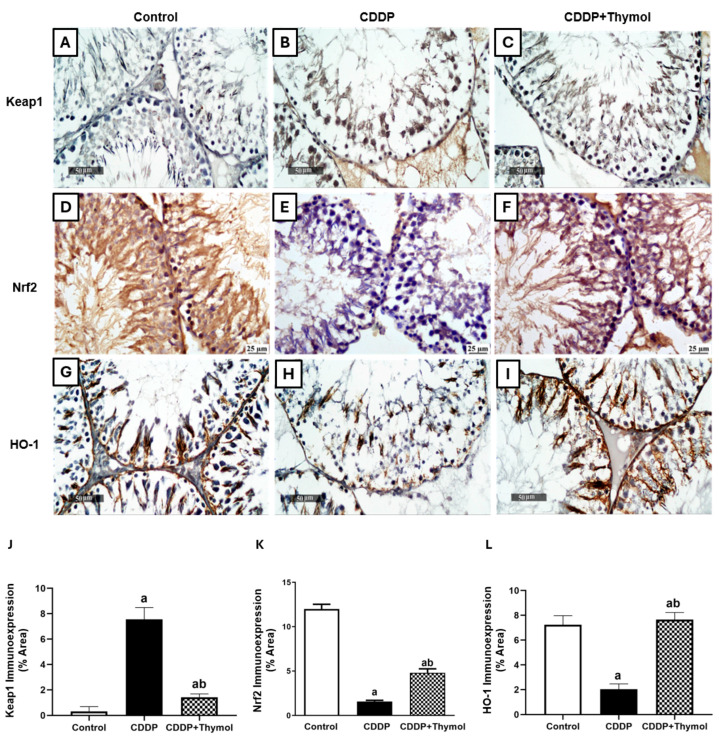
(**A**–**I**) Representative photomicrographs of immunohistochemical staining of Keap1 (**A**–**C**), Nrf2 (**D**–**F**), and HO-1 (**G**–**I**) in rat testicular sections from control, CDDP, and CDDP + thymol groups. Scale bars: 25 µm and 50 µm as indicated. (**J**–**L**) Quantification of immunoexpression (area %) for Keap1, Nrf2, and HO-1, respectively. Data are presented as mean ± SD (n = 6). a and b indicate significant differences compared to the control and CDDP groups, respectively (*p* < 0.05; ANOVA followed by Tukey-Kramer post hoc test). CDDP: cisplatin; Keap1: Kelch-like ECH-associated protein 1; Nrf2: nuclear factor erythroid 2–related factor 2; HO-1: heme oxygenase-1.

To characterize the mechanisms underlying thymol’s protective effects, we examined the expression of key regulators in the antioxidant response pathway, including Keap1, Nrf2, and HO-1 (Figure 5A–L).

CDDP treatment led to a dramatic increase in Keap1 expression, rising by approximately 37-fold compared to control levels, indicating enhanced degradation of Nrf2 and suppression of endogenous antioxidant defenses. Thymol co-administration substantially downregulated Keap1 levels by approximately 82% compared to the CDDP group (Figure 5A–C,J).

Nrf2 immunoexpression was significantly diminished by CDDP, decreasing by approximately 90% compared to the control group. Co-treatment with thymol resulted in a partial recovery of Nrf2 levels, with 2.9-fold increase relative to CDDP alone (Figure 5D–F,K).

HO-1 expression was also notably suppressed by CDDP, with levels reduced by 72% compared to control. Thymol co-treatment increased HO-1 expression by a 3.8-fold over CDDP alone and a slight elevation above baseline (Figure 5G–I,L).

These findings collectively suggest that thymol’s protective effect involves partial reactivation of the Keap1/Nrf2/HO-1 axis, attenuating oxidative stress through modulation of key antioxidant signaling components.

### 3.8. Thymol Inhibits Ferritinophagy-Related Genes mRNA Expression and Reduces Iron-Mediated Oxidative Damage

**Figure 6:**
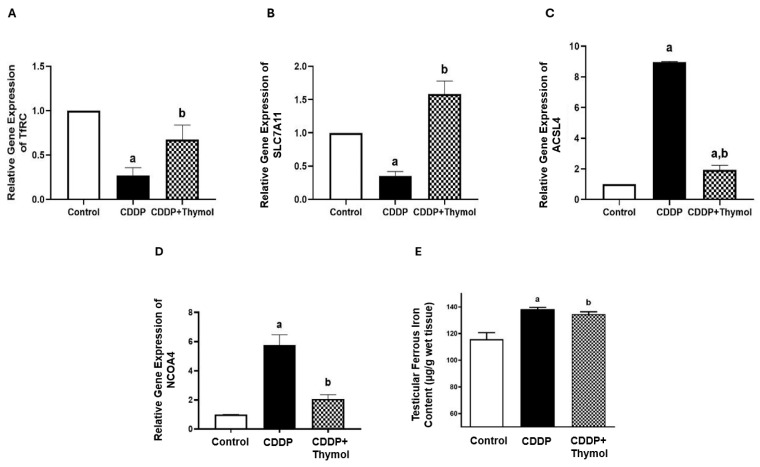
Effect of Thymol and CDDP on Ferritinophagy-Related Gene Expression and Serum Ferrous Levels. (**A**–**D**) Thymol treatment upregulated the mRNA expression of TfRC (**A**) and SLC7A11 (**B**), and downregulated the expression of ACSL4 (**C**) and NCOA4 (**D**) in the testes of rats exposed to CDDP. (**E**) Thymol also reduced the elevated serum ferrous levels induced by CDDP. Data are presented as mean ± SD for gene expression (n = 3) and as mean ± SD for ferrous levels (n = 5). a and b indicate statistically significant differences compared to the control and CDDP groups, respectively (*p* < 0.05, ANOVA followed by Tukey–Kramer post hoc test). CDDP: cisplatin; TfRC: transferrin receptor 1; SLC7A11: cystine/glutamate antiporter; ACSL4: acyl-CoA synthetase long-chain family member 4; NCOA4: nuclear receptor coactivator 4.

To investigate thymol’s role in modulating iron metabolism, we assessed the expression of ferritinophagy-related genes and ferrous ion levels. CDDP treatment significantly suppressed the mRNA expression of both TfRC (–3.7 fold) and SLC7A11 (–2.8 fold), indicating compromised antioxidant capacity. Thymol co-treatment restored the expression of both TfRC (2.5-fold increase) and SLC7A11 (4.5-fold increase) compared to the CDDP group, supporting its role in enhancing cellular antioxidant defenses (Figure 6A,B).

Additionally, CDDP markedly upregulated ACSL4 by approximately 9-fold. Thymol co-treatment significantly reduced ACSL4 expression to about 21% of the CDDP group (Figure 6C). NCOA4 expression also increased by approximately 5.8-fold in the CDDP group compared to control, while thymol co-treatment downregulated it to about 35% of the CDDP group (Figure 6D).

Consistent with these findings, CDDP significantly elevated ferrous ion concentrations by about 20%. Thymol co-treatment reduced ferrous ion levels and inhibited NCOA4-mediated ferritinophagy, limiting the accumulation of free iron and protecting against iron-mediated oxidative damage (Figure 6E).

### 3.9. Thymol Upregulates GPx4, SOD2, and TfR1 Protein Expression in Testicular Tissues

**Figure 7:**
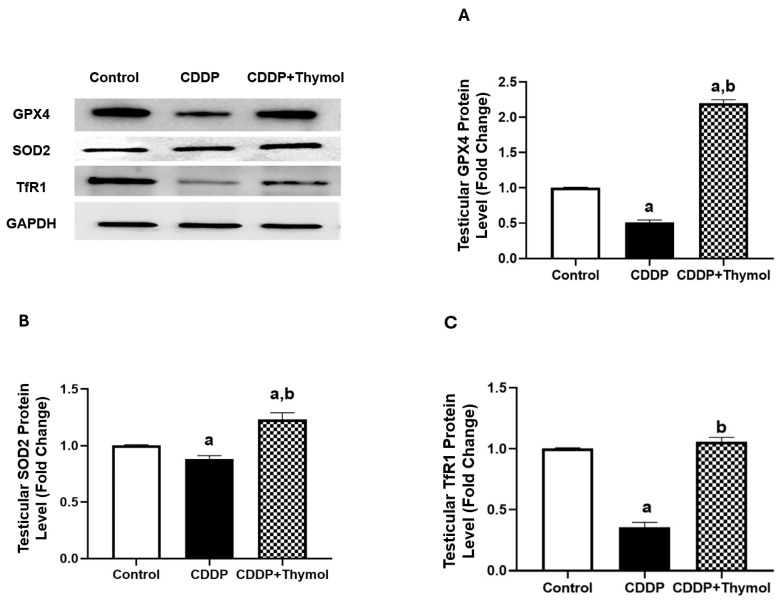
Effects of Thymol and CDDP on Testicular Protein Expression of GPX4, SOD2, and TfR1 in Rats. Protein levels of (**A**) GPX4, (**B**) SOD2, and (**C**) TfR1 were quantified by immunoblotting and normalized to GAPDH. Data are expressed as fold change relative to the control group. Values are mean ± SD (n = 3). a, b: Significantly different from the control and CDDP groups, respectively, at *p* < 0.05 (ANOVA followed by Tukey-Kramer post hoc test). CDDP: cisplatin; GPX4: glutathione peroxidase 4; SOD2: superoxide dismutase 2; TfR1: transferrin receptor 1. Original uncropped Western blot images are provided in Supplementary Figure S5.

To further explore mechanisms of thymol protection in CDDP-induced testicular injury, the expression of downstream ferroptosis- related proteins, SOD2 and GPX4 was evaluated in testicular tissues. CDDP treatment significantly downregulated GPX4 protein expression by approximately 50% compared to control, while thymol co-treatment restored and even enhanced GPX4 levels to about 2.2-fold of the control group (Figure 7A). Similarly, SOD2 expression was reduced by approximately 10% with CDDP treatment, and thymol co-treatment increased its levels to approximately by approximately 20% relative to control (Figure 7B).

Additionally, CDDP led to a sharp reduction of TfR1 protein expression by approximately 65%, whereas thymol co-treatment restored TfR1 levels to control level (Figure 7C).

These findings support the role of thymol in restoring antioxidant defense and preserving iron homeostasis in the context of CDDP-induced ferroptosis and oxidative damage.

### 3.10. Thymol Potentiates CDDP Anticancer Activity and Selectivity

To further assess thymol’s potential in modulating CDDP toxicity, we explored its cytotoxicity profile and selectivity in cancerous and non-cancerous cell lines. Thymol exhibited a moderate cytotoxic effect in the breast cancer MCF-7 cells, with a half-maximal inhibitory concentration (IC_50_) value of 108.35 ± 15.48 µM. In contrast, its toxicity to the non-cancerous MCF-10A cells was minimal, as indicated by an IC_50_ exceeding 1000 µM, which prevented calculation of a Selectivity Index (SI) for thymol (Table 5).

CDDP demonstrated high cytotoxic effects in both MCF-7 and MCF-10A cells (IC_50_ of 5.35 ± 1.54 µM and 6.23 ± 0.04 µM, respectively). The calculated Selectivity Index (SI) of 1.16 indicates limited cancer cell selectivity.

**Figure 8:**
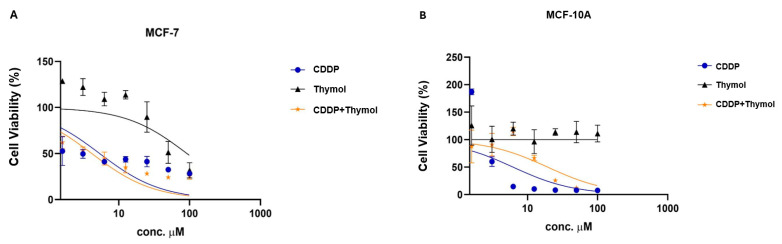
Effect of Thymol on Cisplatin Cytotoxicity in Human Breast Cancer Cells (MCF-7) and Noncancerous Breast Epithelial Cells (MCF-10A). Cell viability was assessed using the MTT assay after treatment with CDDP, Thymol, or their combination across a range of concentrations (0.1–100 µM). (**A**) MCF-7 cells; (**B**) MCF-10A cells. Data are presented as mean ± SD (n = 6). CDDP: cisplatin.

Co-treatment with thymol significantly enhanced CDDP’s cancer cell selectivity. The IC_50_ for MCF-7 and MCF-10A were 4.29 ± 1.58 and 19.39 ± 1.65 µM, respectively, resulting in a four-fold increase in selectivity (SI of 4.51 for the combination compared to 1.16 for CDDP only; Table 5 and Figure 8A,B).

The table shows the IC_50_ values (in μM), reflecting the cytotoxic activity of CDDP, thymol, and their combination in MCF-7 and non-cancerous MCF10A cells, along with the selectivity index (SI). Data are expressed as mean ± SD (n = 6). ND: not detected. CDDP: cisplatin; MCF-10A: non-cancerous breast epithelial cell line; MCF-7: human breast cancer cell line.

## 4. Discussion

CDDP is a cornerstone chemotherapeutic agent, with therapeutic applications in a broad range of solid and hematologic malignancies, such as bladder, breast, and ovarian cancers, as well as Hodgkin and non-Hodgkin lymphoma [57,58,59,60,61,62,63,64,65,66]. However, its use is often limited by toxicity to multiple organ systems, including nephrotoxicity, neurotoxicity, and ototoxicity [1,3,5,14,15,67,68,69]. Its reproductive toxicity extends to both sexes, with potential long-term consequences. In males, CDDP disrupts spermatogenesis, often resulting in irreversible oligospermia or azoospermia. The potential irreversibility of the damage necessitates targeted protective strategies to preserve fertility without compromising antitumor efficacy [13,70,71].

Our study demonstrates that thymol exhibits a protective effect against CDDP-induced testicular toxicity, as evidenced by restored sperm count and viability, along with the preservation of sperm morphology and testicular architecture. These effects were associated with an increase in the levels of testosterone and LH. Thymol has demonstrated antioxidant and anti-inflammatory effects across multiple organ systems and toxic insults [72], such as 5-fluorouracil-induced intestinal mucositis [30,31], dimethylhydrazine- and high-fat diet–induced colon carcinogenesis [51], and gamma irradiation–induced acute nephropathy [73]. In our study, we identify ferritinophagy inhibition as a novel mechanism contributing to thymol’s protective effects against CDDP-induced testicular toxicity. While additional dose-response studies are warranted, our findings highlight a mechanistic link that may inform future translational investigations.

Our findings, consistent with published literature, indicate that CDDP exposure leads to significant testicular histopathological alterations, including a reduction in seminiferous tubule area and volume, loss of key spermatogenesis stages and increased necrotic foci. Many studies have reported a subsequent reduction in testicular weight following CDDP exposure [52,74,75]. However, our results did not show a measurable testicular weight reduction, similar to the findings of Favareto et al. [76]. This discrepancy may arise from differences in study design, including the dosage and duration of CDDP administration, species- or strain-specific resistance to weight loss, or compensatory mechanisms such as interstitial edema, fibrosis, or inflammatory cell infiltration that may offset the expected reduction in testicular weight due to severe histopathological damage.

Despite the preservation of testicular weight, our findings indicate extensive structural and functional damage. The Johnsen’s scoring system further confirmed severe germ cell maturation failure in CDDP-treated animals, with a mean score of 5, indicating extensive spermatogenic disruption. Similar gross and histopathological alterations have been reported following CDDP treatment, including germ and Leydig cell apoptosis, structural disintegration of testicular architecture, and intertubular edema [49,77,78]. Histological alterations were accompanied by corresponding sperm abnormalities. CDDP treatment induced significant oligospermia, consistent with findings reported by other groups [12,79,80]. In line with this, we observed reduced viability in the CDDP-treated group. The structural and functional impairments in the CDDP-treated animals were accompanied by endocrine disturbances, as evidenced by reduced testosterone and LH levels, suggesting impaired steroidogenesis and Leydig cell dysfunction.

Thymol co-administration significantly improved testicular function, as evidenced by the restoration of spermatogenesis stages and an increase in Johnsen’s scores to 9, nearing control levels. Although structural recovery of seminiferous tubule volume and area was limited, the reestablishment of spermatogenic activity reflects near-complete functional restoration. Co-administration of thymol with CDDP salvaged total sperm count and preserved sperm morphology, further supporting its protective effects on the reproductive function. Beyond its role in mitigating CDDP-induced toxicity, thymol alone significantly enhanced sperm viability, with a modest increase in sperm count. This finding suggests a broader role for thymol in promoting male fertility, independent of its chemotherapy-protective effects. The structural and functional recovery provided by thymol co-treatment is further supported by the normalization of LH and testosterone levels, suggesting that thymol may protect Leydig cell function.

CDDP-induced testicular toxicity is driven by multiple interrelated mechanisms, primarily oxidative stress and inflammation, which culminate in lipid peroxidation, DNA damage, and apoptosis [81,82,83,84]. Our findings confirm that CDDP significantly elevated MDA levels while depleting key antioxidant defenses, including SOD, and GPX, highlighting the role of oxidative stress as a central driver of testicular toxicity.

The role of Nrf2 in regulating antioxidant cellular defenses has recently been an area of active investigation. Under low oxidative stress conditions, Nrf2 remains sequestered in the cytoplasm by Keap1, which promotes its ubiquitination and subsequent proteasomal degradation. In response to ROS, the oxidative stress sensor Keap1 undergoes structural modifications, triggering Nrf2 release, stabilization, and translocation into the nucleus. Once inside the nucleus, Nrf2 functions as a transcription factor, binding to antioxidant response elements (AREs) to induce the expression of HO-1 along with other antioxidant genes. HO-1 activity promotes the generation of carbon monoxide (CO) and Fe^2+^, which in turn stimulate ferritin synthesis, essential for iron storage and homeostasis [85]. Our findings confirm that CDDP treatment upregulated Keap-1 expression, while downregulated the Nrf2/HO-1 pathway, exacerbated oxidative stress, and impaired the cellular defense response. Thymol restored the antioxidant balance, enhanced antioxidant enzyme activity, reduced lipid peroxidation, and upregulated Nrf2 and HO-1, while suppressing Keap1 expression. These findings align with other reports on other antioxidant-based interventions, such as lycopene, N-acetylcysteine [49], daflon [24], taurine [78], and quercetin [81], all of which have been shown to mitigate CDDP-induced oxidative stress through enhancing antioxidant defenses.

Our data demonstrate that CDDP led to an upregulation of pro-inflammatory cytokines, particularly TNF-α, contributing to testicular inflammation. In contrast, we observed a significant reduction in IL-6 levels in the testes of CDDP-treated animals, and this effect was reversed by thymol administration (Table 4). This paradox may reflect the complex and context-dependent nature of IL-6, which can mediate either pro- or anti-inflammatory responses depending on whether it signals through its membrane-bound or soluble receptor forms [86]. In the testes, IL-6 has also been implicated in regulating Sertoli cell function and germ cell activity [87]. Our results indicate a reduction in IL-6 levels, which may reflect CDDP’s toxic effects on local IL-6–producing cells or a tissue-specific regulatory response, though the exact mechanism remains unclear.

Furthermore, the interplay between oxidative stress and inflammation likely exacerbates testicular injury. Excessive ROS has been shown to activate NF-κB signaling in CDDP-induced organ toxicities, further amplifying pro-inflammatory cytokine expression [6]. By modulating both oxidative stress and inflammatory pathways, thymol provides dual protection against CDDP-induced toxicity.

Recent studies suggest that ferritinophagy and ferroptosis play a pivotal role in chemotherapy-induced toxicity. Ferritinophagy, a selective autophagy process, degrades ferritin via the cargo receptor NCOA4, releasing Fe^2+^ into the labile iron pool (LIP). While ferritinophagy is essential for iron homeostasis, excessive ferritin degradation under stress conditions leads to iron overload, exacerbating oxidative stress through the Fenton reaction and triggering ferroptotic cell death [16].

Our results confirm that CDDP exposure upregulated key ferritinophagy markers, including NCOA4, while significantly increasing free iron levels and ACSL4 expression, a key enzyme involved in lipid peroxidation and ferroptosis. These changes suggest that CDDP-induced oxidative stress drives excessive ferritin degradation, increasing Fe^2+^ release, which amplifies ROS accumulation and exacerbates cellular damage.

The crosstalk between the Nrf2/HO-1 pathway and ferritinophagy is central to iron homeostasis and oxidative stress regulation. HO-1 not only functions as an antioxidant enzyme but also plays a key role in iron metabolism by promoting ferritin synthesis, thereby sequestering excess Fe^2+^ and preventing its participation in Fenton reactions. However, CDDP-induced suppression of Nrf2 and downregulation of HO-1 impair this protective mechanism, leading to dysregulated ferritin turnover and iron overload [17].

Thymol co-treatment counteracted these effects by restoring Nrf2 activity, upregulating HO-1, and limiting NCOA4-driven ferritinophagy, thereby reducing Fe^2+^ accumulation. Additionally, thymol downregulated ACSL4 expression, suppressing lipid peroxidation and ferroptosis progression. The restoration of SLC7A11 expression, a key component of the GSH-dependent antioxidant system, further reinforces thymol’s role in mitigating ferroptosis and preserving testicular integrity.

A significant limitation of CDDP therapy is its poor selectivity, leading to toxicity in normal tissues. Our in vitro findings demonstrate that thymol alone exhibited moderate cytotoxicity in MCF-7 breast cancer cells while displaying markedly lower toxicity in non-cancerous MCF-10A cells (IC_50_ > 1000 µM). While CDDP alone showed limited selectivity (SI = 1.16), thymol co-treatment significantly enhanced cancer selectivity (SI = 4.51), indicating that thymol may improve the therapeutic index of CDDP by protecting normal cells while maintaining anticancer efficacy.

By modulating oxidative stress, inflammation, and iron homeostasis, thymol emerges as a promising candidate for fertility preservation in cancer patients receiving CDDP therapy. Further investigations are needed to optimize its therapeutic application as an adjunct to chemotherapy, potentially minimizing off-target toxicities while preserving reproductive function.

This study evaluated the effects of a single tested dose of thymol in a CDDP-induced testicular toxicity model. While this approach demonstrated protective potential, future studies incorporating dose–response analyses would help define the therapeutic range. Additionally, the findings were based on analyses at a single time point, which provided a snapshot of testicular response but did not capture longer-term outcomes. Time-course studies and complementary in vitro work may further clarify the long-term effects and underlying mechanisms. These directions will help advance its translational relevance as a potential chemoprotective agent.

## 5. Conclusions

This study provides preliminary evidence that thymol, a natural compound with a well-established safety profile, may protect against CDDP-induced testicular damage, offering a promising option for cancer patients undergoing chemotherapy. The observed protective effects appear to involve multiple mechanisms, including reduction of oxidative stress and inflammation, inhibition of ferritinophagy, and enhancement of CDDP selectivity toward cancer cells. These findings suggest thymol’s potential as a fertility-preserving adjunct during chemotherapy; however, further studies using multiple doses and time points are warranted to validate and expand upon these results.

## Figures and Tables

**Table 1:** Primer sequences of target genes used for RT-PCR.

Gene	Primer Sequence (5′ → 3′)	Accession Number
*ACSL4*	Forward: TCACCATTGTATTGCTGCCT Reverse: GAGCGATATGGACTTCCG	NM_053623.1
*TFRC*	Forward: ATCAAGCCAGATCAGCAT Reverse: GGGTTTTCTGACACTAGC	NM_022712
*SLC7A11*	Forward: GAGCCACCTGGGCATGAGAA Reverse: CCACAGGCAGACCAGAACAC	NM_001107673
*17β-HSD*	Forward: CAACCTGCTCCCAAGTCA Reverse: AACCCCTACTCCCGAAGA	NM_054007.1
*3β-HSD*	Forward: GGTGCAGGAGAAAGAACT Reverse: GGCATCCAGAATATCTCC	NM_001007719.3
*StAR*	Forward: TTGGGCATACTCAACAACCA Reverse: CACCAGTTCTTCATAGAGTC	NM_031558
*NCOA4*	Forward: ACGCGAGCTCCTCAAGTATT Reverse: AGTCCTGTGGGTTGGTACTG	NM_019744

*ACSL4*: acyl-CoA synthetase long-chain family 4; *TFRC*: transferrin receptor 1 (*Tfr1*) gene; *SLC7A11*: cystine/glutamate antiporter; *17β-HSD*: 17β-Hydroxysteroid dehydrogenase; *3β-HSD:* 3β-Hydroxysteroid dehydrogenase; *StAR*: steroidogenic acute regulatory protein; *NCOA4*: nuclear receptor coactivator 4.

**Table 2:** Binding Energies and Molecular Interactions of Thymol with HO-1 and ACSL4.

Protein	Binding Energy (Kcal/mol)	Amino Acid Residues of Interaction	Types of Bonds (Distances Å)
HO-1	−4.63	Arg 136	H-bond (2.18)
		Ala 28	Pi-alkyl (4.73)
		Phe 207	Pi-alkyl (4.42)
		Phe 214	Pi-alkyl (4.89)
ACSL4	−4.76	Glu 470	H-bond (2.52)
		Leu 468	Pi-alkyl (5.28)
		Lys 690	Pi-alkyl (5.26)
		Leu 690	Pi-cation (4.91)

HO-1: heme oxygenase-1; ACSL4: acyl-CoA synthetase long-chain family 4.

**Table 3:** Effects of Thymol and CDDP on Body Weight, Testicular Weight, and Serum Levels of Testosterone and LH.

	Body Weight Changes (g)	Testis Weight (g)	Testosterone (ng/mL)	LH (mIU/mL)
Control	17.89 ± 4.56	1.6 ± 0.17	7.86 ± 0.55	79.69 ± 7.09
CDDP (8 mg/kg)	−41.00 ± 17.59 ^a^	1.62 ± 0.16	3.81 ± 0.30 ^a^	23.49 ± 3.31 ^a^
CDDP (8 mg/kg) + Thymol (60 mg/kg)	−7.50 ± 8.01 ^a,b^	1.43 ± 0.32	6.07 ± 0.70 ^a,b^	64.9 ± 4.56 ^a,b^
Thymol (60 mg/kg)	15.25 ± 3.01 ^b^	1.73 ± 0.20	7.57 ± 0.63 ^b^	75.06 ± 7.31 ^b^

Data are expressed as mean ± SD, n = 5. ^a,b^ indicate statistical differences from the control and CDDP groups, respectively (*p* < 0.05) using ANOVA followed by Tukey-Kramer as a post hoc test. CDDP: cisplatin; LH: luteinizing hormone.

**Table 4:** Effect of Thymol and CDDP on Oxidative Stress and Inflammatory Markers.

	MDA (nmol/g Tissue)	SOD (U/g Tissue)	GPX (U/g Tissue)	TNF-α (pg/mL)	IL-6 (pg/mL)
Control	111 ± 9.86	104.0 ±17.82	1.80 ± 0.09	33.19 ± 3.51	59.69 ± 5.84
CDDP (8 mg/kg)	210 ± 7.50 ^a^	57.00 ± 4.47 ^a^	1.10 ± 0.07 ^a^	62.75 ± 7.71 ^a^	34.91 ± 2.89 ^a^
CDDP (8 mg/kg) + Thymol (60 mg/kg)	187 ± 12.40 ^a,b^	77.00 ± 2.09 ^a,b^	1.71 ± 0.15 ^a,b^	42.0 ± 1.69 ^a,b^	44.52 ± 4.21 ^a,b^
Thymol (60 mg/kg)	101 ± 12.1 ^b^	107.4 ± 8.23 ^b^	1.56 ± 0.25 ^b^	35.29 ± 6.37 ^b^	55.40 ± 4.56 ^b^

Data were expressed as mean ± SD (n = 5). ^a,b^ indicate statistical differences from the control and CDDP groups, respectively (*p* < 0.05), using ANOVA followed by Tukey-Kramer as a post hoc test. CDDP: cisplatin; MDA: malondialdehyde; SOD: superoxide dismutase; GPX: glutathione peroxidase; TNF-α; tumor necrosis factor-α; IL-6: interleukin-6.

**Table 5:** Effect of Thymol on the Anticancer Activity and Cytotoxicity of Cisplatin.

Treatment	IC_50_ MCF-7	IC_50_ MCF10A	Selectivity Index (SI)
CDDP	5.35 ± 1.55	6.23 ± 04	1.16
Thymol	108.35 ± 15.48	>1000	ND
CDDP + Thymol	4.29 ± 1.58	19.43 ± 1.65	4.51

## Data Availability

The authors confirm that the data supporting the findings of this study are available within the article.

## Supplementary Materials

The following supporting information can be downloaded at: https://www.mdpi.com/article/10.3390/biom15091277/s1, Figure S1: Molecular docking visualization showing the binding interactions between thymol and key residues at the HO-1 binding site; Figure S2: Binding site representation of HO-1; Figure S3: Binding interactions of thymol at the ACSL4 binding site; Figure S4: Binding pocket of ACSL4 showing interacting residues surrounding thymol; Figure S5: Original Western blots showing testicular protein expression of GPX4, SOD2 (with GAPDH as loading control), and TfR1/CD71 in control, cisplatin-treated, and cisplatin + thymol-treated rats.
