# Supplementary material for: Thymol Preserves Spermatogenesis and Androgen Production in Cisplatin-Induced Testicular Toxicity by Modulating Ferritinophagy, Oxidative Stress, and the Keap1/Nrf2/HO-1 Pathway
Source: Biomolecules. 2025 Sep 3;15(9):1277. doi: 10.3390/biom15091277 (PMC12467222; doi:10.3390/biom15091277)
Supplement: Supplementary file 1 [file biomolecules-15-01277-s001.zip › biomolecules-3699549-supplementary.pdf]

Supplementary Materials

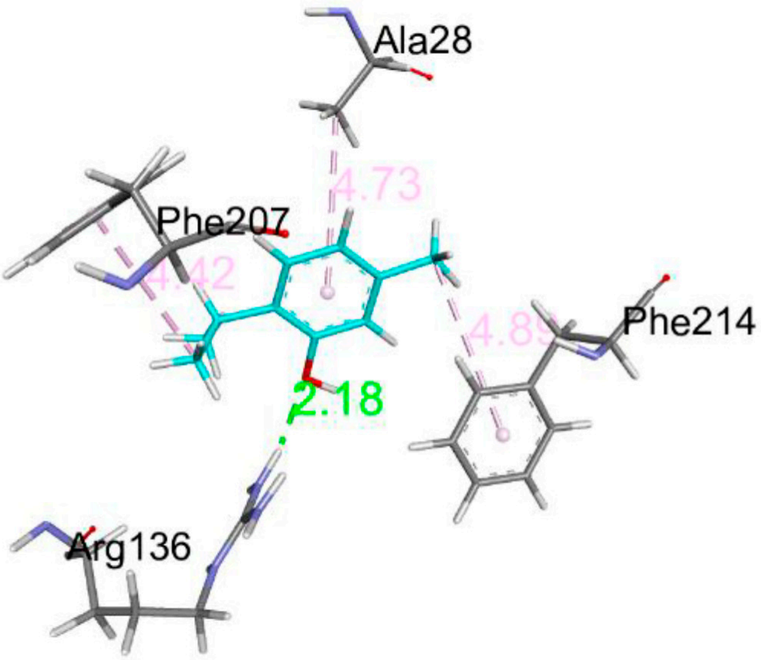

Supplementary Figure S1. Molecular docking visualization showing the binding interactions between thymol and key residues at the HO-1 binding site. Distances (in Å) between thymol and surrounding amino acid residues (Ala28, Phe207, Phe214, Arg136) are indicated.

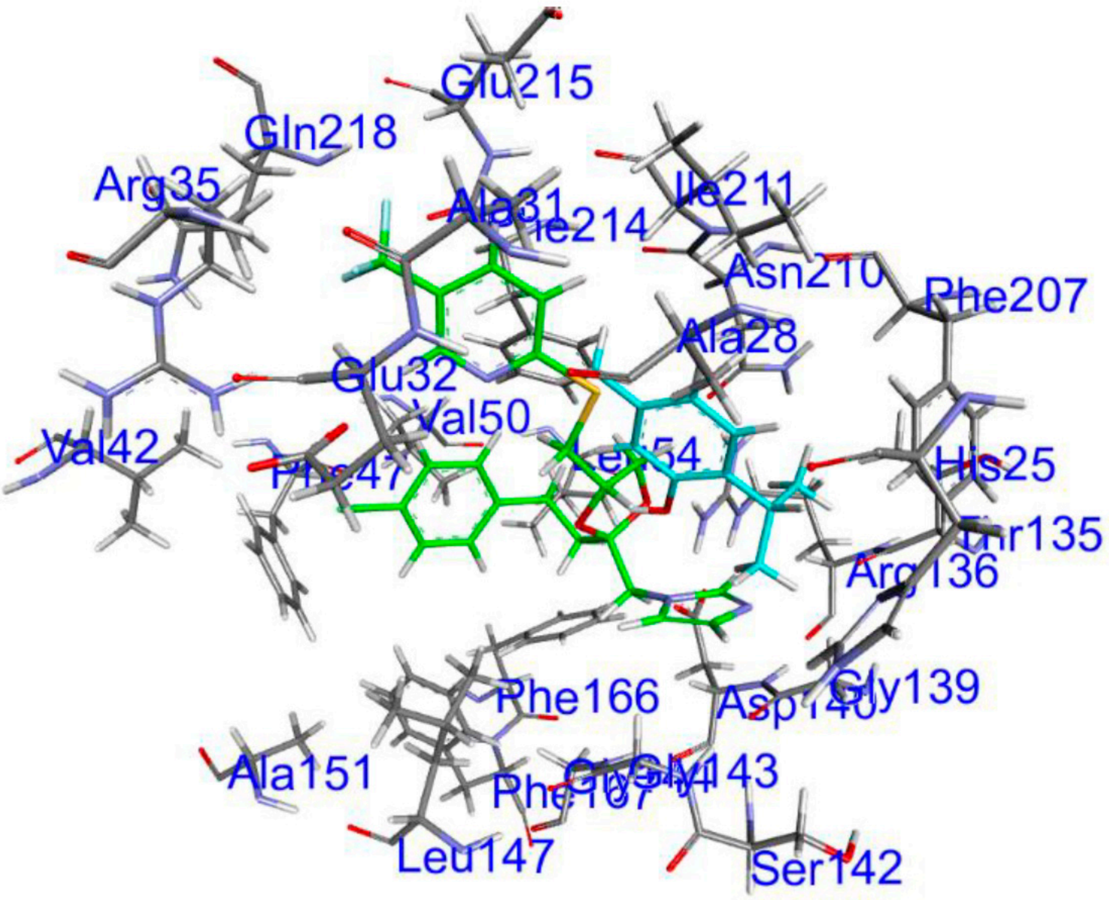

Supplementary Figure S2. Binding site representation of HO-1 showing the co-crystallized ligand QC-80 (green sticks), which defines the active site residues used for docking. Thymol (blue sticks) is overlaid within the same binding pocket.

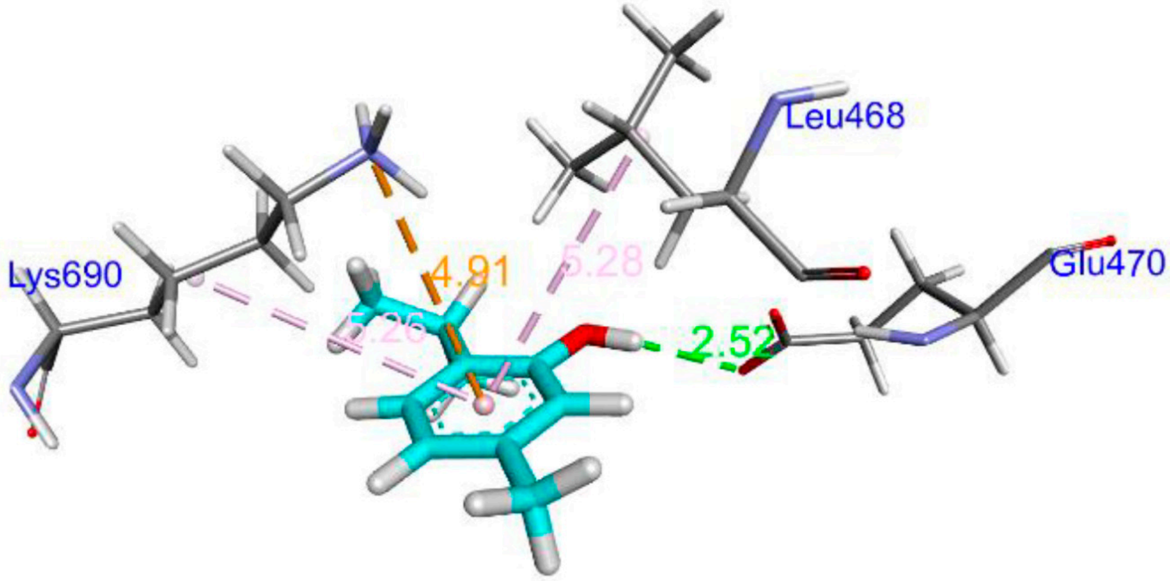

Supplementary Figure S3. Binding interactions of thymol at the ACSL4 binding site. Thymol is shown in cyan sticks, with key interacting residues (Lys690, Leu468, Glu470) depicted in gray.

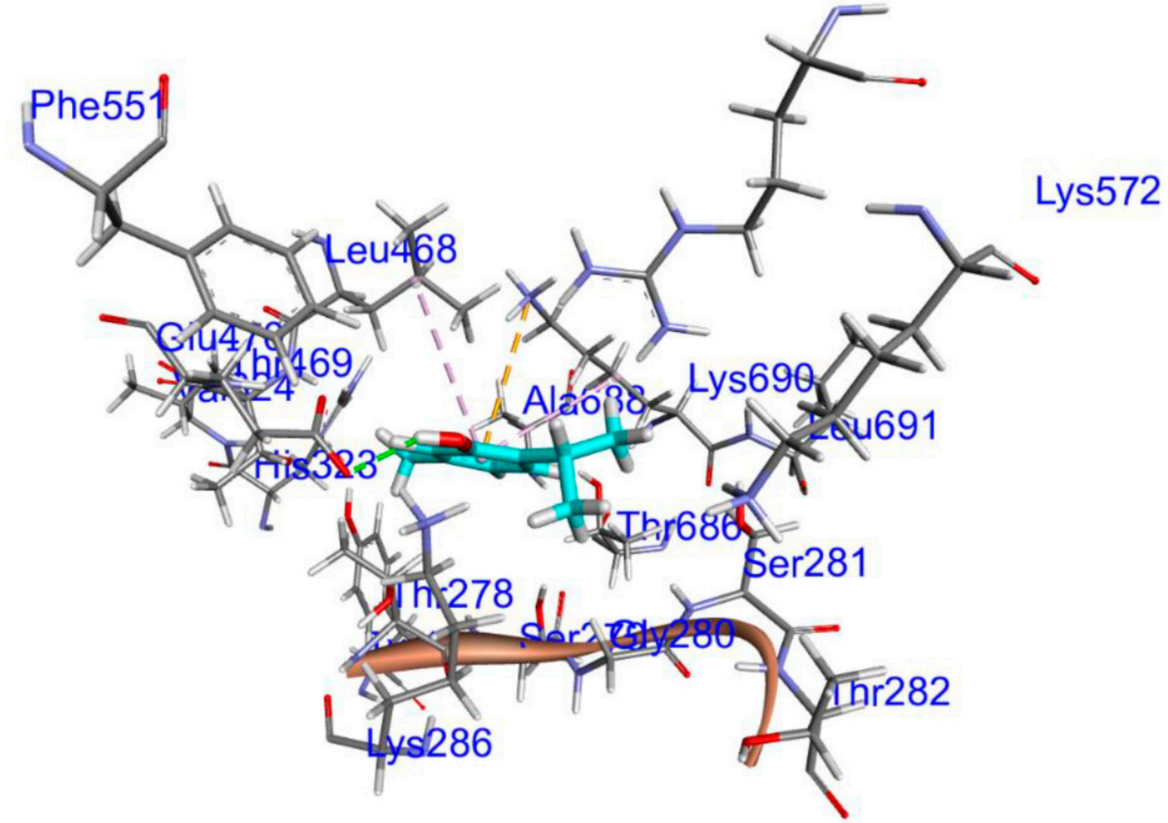

Supplementary Figure S4. Binding pocket of ACSL4 showing interacting residues surrounding thymol. Thymol is depicted in cyan sticks, with key amino acids in the binding pocket labeled.

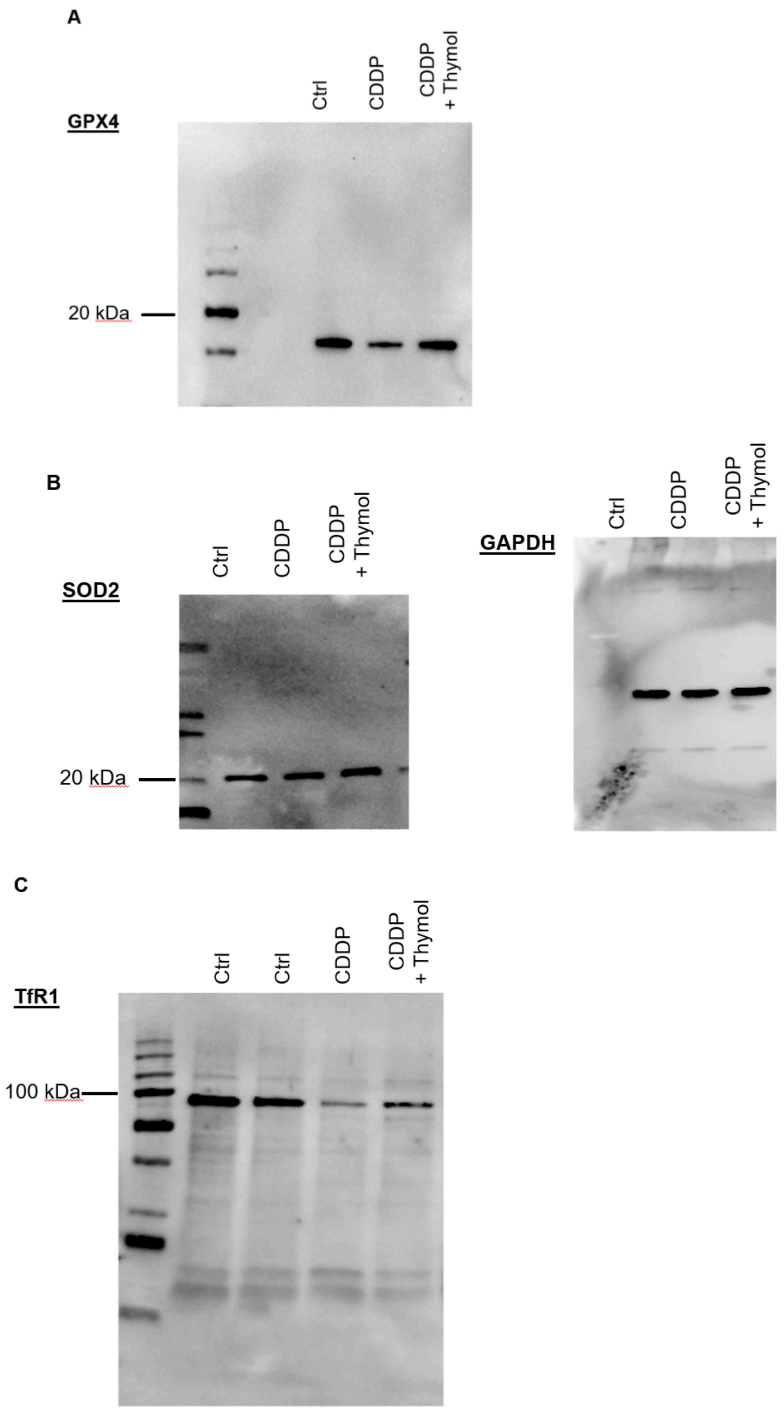

Supplementary Figure S5. Original Western blot images corresponding to Figure 7 in the main text. Protein expression of (**A**) GPX4 (~20 kDa), (**B**) SOD2 (~22 kDa) with GAPDH (~37 kDa) as the loading control, and (**C**) TfR1/CD71 (~95–100 kDa) in testicular tissue from control (Ctrl), cisplatin-treated (CDDP), and cisplatin + thymol-treated rats. Molecular weight markers are indicated.
